# Age- and sex-specific patterns of microcytic anemia in adolescence: implications for screening

**DOI:** 10.1007/s00431-026-07279-6

**Published:** 2026-08-04

**Authors:** Tal Ben-Ami, Avigail Eisenberg-Wygoda, Shoshana Revel-Vilk

**Affiliations:** 1 Pediatric Hematology Unit, Kaplan Medical Center, 1 Pasternak St., Rehovot, 7610041 Israel; 2 Faculty of Medicine, Hebrew University, Jerusalem, Israel; 3 Department of Pediatric Hematology-Oncology, Ruth Rappaport Children’s Hospital, Rambam Health Care Campus, Haifa, Israel; 4 Pediatric Hematology/Oncology Unit, The Eisenberg R&D Authority, Shaare Zedek Medical Center, Jerusalem, Israel

**Keywords:** Microcytic anemia, Adolescence, Screening, Iron deficiency

## Abstract

To characterize age- and sex-specific patterns of microcytic anemia during adolescence and to estimate the potential yield of screening across age groups. We performed a retrospective Electronic Health Record (EHR)-based study using data from Clalit Health Services (CHS), the largest health maintenance organization in Israel. Adolescents aged 12–18 years with at least one hemoglobin and mean corpuscular volume (MCV) measurement between 2003 and 2023 were included. Individuals with hereditary, hemolytic, or chronic inflammatory causes of anemia were excluded. Microcytic anemia was defined as hemoglobin < 12 g/dL for females and < 13 g/dL for males with MCV < 78 fL. Age- and sex-specific prevalence was calculated among tested adolescents and in the total CHS-insured population. Screening efficiency was assessed using the number needed to test (NNT). A total of 1,306,623 test records were analyzed. Among females, the prevalence of microcytic anemia increased with age, from 7.8% at ages 12–13 to 12.5% at ages 17–18. Among males, prevalence declined from 18.9% at age 12–13 to 2.1% at ages 17–18. This resulted in a reversal of the sex distribution of microcytic anemia prevalence during mid-adolescence, with females exceeding males from approximately age 15 onward. The NNT increased with age among males and remained relatively stable during mid- to late adolescence among females.

*Conclusions*: Microcytic anemia demonstrates distinct age- and sex-specific patterns during adolescence. The increasing prevalence among females and declining prevalence among males suggest that targeted screening of adolescent girls during mid-adolescence may be an efficient strategy for identifying microcytic anemia, a condition commonly associated with iron deficiency in this age group.

**Table:** 

**What is Known:** • *Iron deficiency anemia remains common in adolescents, particularly among females in high-income countries.* • *Prevalence estimates vary widely due to differences in definitions, populations, and study designs.* • *There are no universally accepted guidelines for routine anemia screening in adolescents, and current practices are inconsistent.*	
**What is New:** *• A reversal in sex-specific prevalence of microcytic anemia occurs during mid-adolescence, with higher rates in females from approximately age 15 onward.* • *Screening efficiency, quantified by number needed to test, varies substantially by age and sex, supporting targeted screening of adolescent girls at age 14.*

**What is Known:**

• *Iron deficiency anemia remains common in adolescents, particularly among females in high-income countries.*

• *Prevalence estimates vary widely due to differences in definitions, populations, and study designs.*

• *There are no universally accepted guidelines for routine anemia screening in adolescents, and current practices are inconsistent.*

**What is New:**

*• A reversal in sex-specific prevalence of microcytic anemia occurs during mid-adolescence, with higher rates in females from approximately age 15 onward.*

• *Screening efficiency, quantified by number needed to test, varies substantially by age and sex, supporting targeted screening of adolescent girls at age 14.*

## Introduction

Iron deficiency anemia (IDA), the most common cause of microcytic anemia in adolescents, remains one of the most prevalent nutritional disorders worldwide and continues to pose a major public health challenge across the life course [1–3]. Iron deficiency (ID) is the leading cause of anemia globally and affects an estimated two billion individuals, with a disproportionate burden among infants, children, adolescents, and women of reproductive age [3, 4]. Beyond its hematologic manifestations, ID is associated with impaired neurocognitive development, reduced physical capacity, adverse pregnancy outcomes, and diminished quality of life [5–7].

In high-income countries, ID and IDA are often perceived as conditions largely confined to specific risk groups. However, emerging evidence indicates that they remain major health concerns among adolescents and young adults, particularly females, driven by dietary patterns, menstrual blood loss, and increased physiological demands, and are frequently under-recognized in routine clinical practice [8–11]. Recent US and European population-based studies report ID or IDA in approximately 18–20% of adolescent girls. In contrast, the prevalence of ID and IDA in adolescent boys is substantially lower, often below 5%. Prevalence estimates vary widely depending on the diagnostic definition used and on whether iron status is assessed by ferritin, hemoglobin, or both [12–15].

Despite the availability of effective diagnostic tools and safe, inexpensive treatment, routine screening for ID and IDA in adolescents remains inconsistent across healthcare systems. Variability in ferritin thresholds, uncertainty regarding the clinical significance of subclinical ID, and limited population-level data on age- and sex-specific prevalence rates have contributed to inconsistent screening practices [14, 16, 17, 18]. The recently published American Academy of Pediatrics (AAP) clinical report recommends screening for ID in adolescent girls who are at least 1 year post menarche, but no later than 14 years of age [19]. However, this recommendation has not yet been widely incorporated into clinical practice. This issue is particularly relevant for adolescent girls, in whom the prevalence of ID and IDA is substantially higher than in males and in whom physiological risk factors such as menstruation are predictable and lasting. High-quality population-based data are therefore essential to inform evidence-based decisions about whether and in whom screening may be justified.

Israel provides a unique setting for examining the epidemiology of microcyticanemia. The population is characterized by marked ethnic, socioeconomic, and cultural heterogeneity, as well as universal health insurance coverage and comprehensive electronic medical records. Clalit Health Services (CHS), the largest healthcare organization in Israel, maintains a comprehensive longitudinal EHR database that captures clinical and laboratory data across diverse ethnic, socioeconomic, and cultural groups, providing a unique setting in which to examine the epidemiology of microcytic anemia and its distribution across age and sex-specific strata [20, 21].

Our study used data from the CHS electronic health record (EHR) database to characterize age- and sex-specific trends in the prevalence of microcytic anemia during adolescence and to estimate the potential yield of screening across age groups. Because IDA is a common and clinically important cause of microcytic anemia in this population, these findings were interpreted within that clinical context. However, ferritin-confirmed ID was not directly measured, and microcytic anemia should not be assumed to represent IDA in all cases. By providing population-level estimates across defined age and sex strata within a large national healthcare setting, this analysis aims to better define the burden and distribution of presumed IDA in adolescents. These findings may inform ongoing national and international discussions on optimal screening strategies and the identification of adolescent populations that may benefit most from targeted screening.

## Methods

### Study design and population

We conducted a retrospective EHR-based analysis using deidentified CHS data from January 1, 2003, through December 31, 2023. CHS is the largest integrated health care organization in Israel, which provides care to approximately 4.8 million members nationwide. CHS operates a network of > 1500 community clinics, 14 hospitals, and multiple specialized outpatient services, all linked through a unified EHR system implemented more than two decades ago. The CHS EHR includes longitudinal information on demographics, diagnoses, laboratory test results, prescriptions, and encounters across care settings, enabling comprehensive EHR-based analyses of adolescent health.

Data extraction was performed using Wiser (version alpha, Rehovot), a longitudinal machine learning platform [22, 23]. The study population included adolescent females and males aged 12 to 18 years who had at least one hemoglobin measurement recorded in the CHS laboratory database. Adolescents were excluded if they had a documented diagnosis of chronic anemia, identified using ICD-10-CM codes, including thalassemia (D56), hemolytic anemia (D55-D59), hereditary spherocytosis (D58.0), sickle cell disease (D57), or aplastic anemia (D60-D61), as recorded in the CHS diagnosis registry. Individuals with chronic conditions that may affect hemoglobin levels were also excluded, including celiac disease (K90.0), inflammatory bowel disease (Crohn’s disease K50, ulcerative colitis K51), and malignancy (C00-C96). Date of data extraction was February 13, 2025.

Ethical approval was obtained from the institutional review board (No. KMC-24–0089). Owing to the retrospective design and the use of deidentified data, the requirement for informed consent was waived in accordance with local regulations and applicable ethical guidelines.

### Definition of microcytic anemia

Microcytic anemia was defined a priori using sex-specific hemoglobin and MCV thresholds below the 5th percentile of the CHS laboratory reference range for individuals 12- 18 years of age as follows: hemoglobin < 12 g/dl for females and < 13 g/dl for males, together with mean corpuscular volume (MCV) < 78 fL [24]. Paired hemoglobin and MCV measurements were used to define the microcytic cohort. The uniform MCV threshold was applied across age and sex strata to provide a consistent operational definition for comparative analyses. The analysis was based on aggregated age- and sex-specific counts generated from the EHR query, as the approved ethics protocol did not permit the extraction of individual-level laboratory data. Consequently, continuous red blood cell (RBC) indices were not available for analysis. Only records with both hemoglobin and MCV values from the same laboratory request were included; records missing either value were excluded. No missing data were therefore present for the primary outcome variables.To examine whether the hemoglobin threshold influenced prevalence estimates in early-pubertal males, a sensitivity analysis was performed for boys aged 12–13 years using a threshold of < 12 g/dL, equivalent to the female cutoff.

### Age stratification and denominators

Analyses were performed within six predefined 1-year age strata (12–13, 13–14, 14–15, 15–16, 16–17, and 17–18 years) and were stratified by sex. For each individual, only the first available hemoglobin and MCV measurement within each age stratum was included, yielding a single observation per person per age group. Accordingly, adolescents could contribute up to six observations across the full age range. The unit of analysis was therefore the person-age-stratum record.

Two complementary denominators were used. The primary denominator comprised adolescents who underwent hemoglobin testing within each age-sex stratum, enabling estimation of the prevalence of microcytic anemia among those tested. As a secondary analysis, estimates were calculated using the total number of CHS-insured adolescents in each age-sex stratum as the denominator, providing conservative estimates of the proportion of adolescents with documented microcytic anemia within the CHS-insured population. These estimates should not be interpreted as the true population prevalence because adolescents who were not tested could not be classified.

### Statistical analysis

Age- and sex-specific prevalence rates were calculated and expressed as percentages with corresponding 95% confidence intervals (CI) based on binomial distributions and as cases per 1,000 individuals. The number needed to test (NNT) to identify one case of microcytic anemia was derived as the inverse of the age- and sex-specific prevalence among tested adolescents. All analyses were conducted using R software (R Foundation for Statistical Computing, Vienna, Austria).

## Results

### Study population

**Fig. 1:**
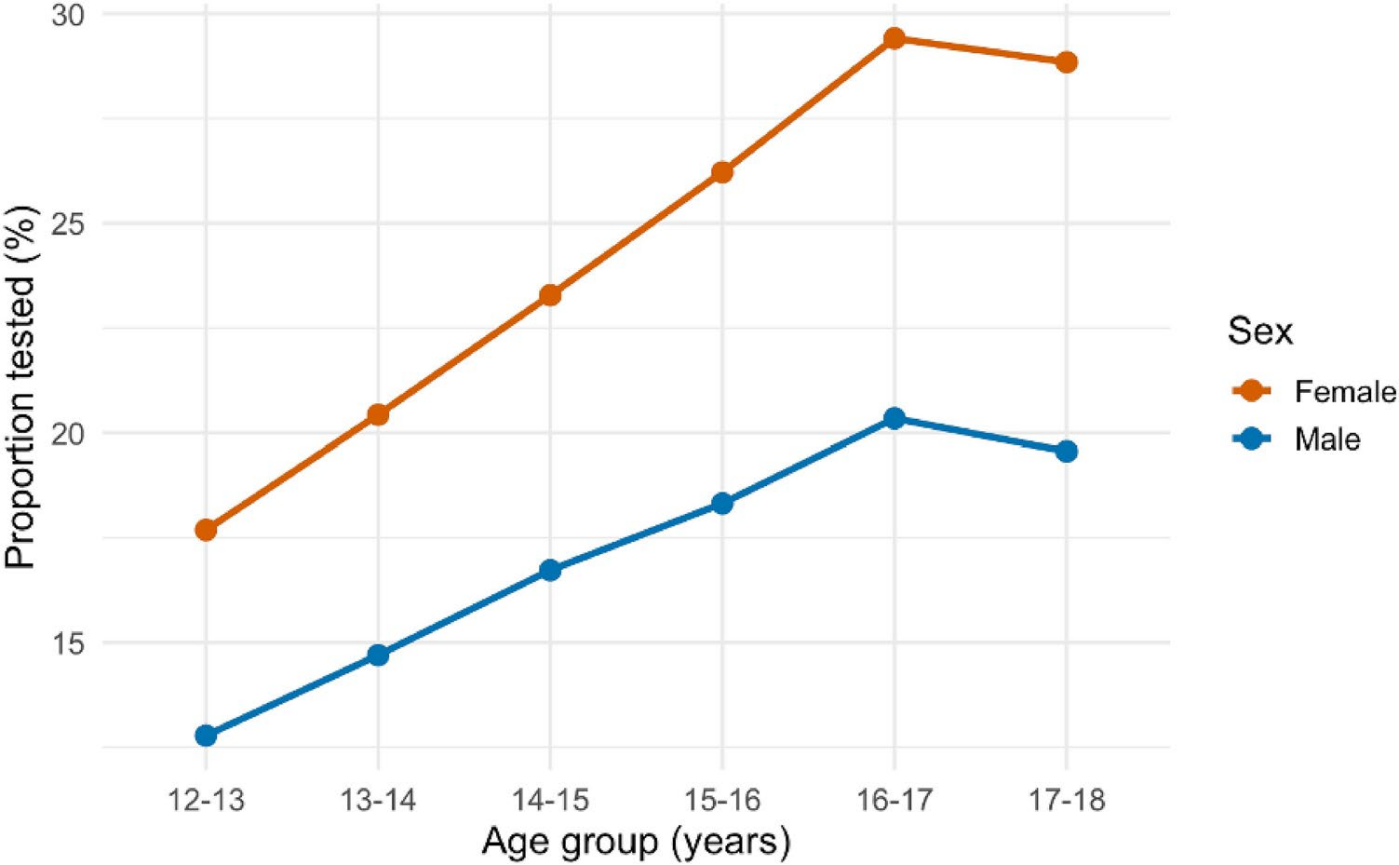
Proportion of individuals with a recorded hemoglobin test by age group and sex

A total of 1,306,623 hemoglobin and MCV testing records were available for analysis, representing 13–29% of the insured population for each age group. The total insured adolescent population within CHS was stable across age groups, comprising approximately 500,000 to 535,000 females and 530,000 to 565,000 males per single-year age stratum. The proportion of individuals with hemoglobin and MCV testing increased progressively with age in both sexes (Fig. 1), with consistently higher testing rates among females compared with males.

Points represent the percentage of the population tested within each age group, and lines illustrate trends across age categories.

### Prevalence of microcytic anemia among tested individuals

Marked differences in the prevalence of microcytic anemia were observed according to sex and age (Table 1).

**Table 1:** Prevalence of microcytic anemia and NNT among adolescents with a recorded hemoglobin measurement, by age group and sex

Age group (years)	Sex	Microcytic anemia cases, *n*	Adolescents tested, *n*	Prevalence, % (95% CI)	NNT
12–13	Female	7389	94,516	7.8 (7.6–8.0)	12.8
	Male	13,666	72,180	18.9 (18.6–19.2)	5.3
13–14	Female	10,683	107,402	9.9 (9.8–10.1)	10.1
	Male	11,831	81,796	14.5 (14.2–14.7)	6.9
14–15	Female	13,831	120,505	11.5 (11.3–11.7)	8.7
	Male	8,068	91,793	8.8 (8.6–9.0)	11.4
15–16	Female	15,982	133,778	11.9 (11.8–12.1)	8.4
	Male	4,996	99,274	5.0 (4.9–5.2)	19.9
16–17	Female	18,195	148,449	12.3 (12.1–12.4)	8.2
	Male	3,197	109,081	2.9 (2.8–3.0)	34.1
17–18	Female	17,982	144,149	12.5 (12.3–12.6)	8.0
	Male	2,175	103,700	2.1 (2.0–2.2)	47.7

*NNT* number needed to test. Prevalence was calculated as the proportion of adolescents with recorded hemoglobin and MCV measurements who met the criteria for microcytic anemia within each age-sex stratum. Confidence intervals were calculated using the Wilson method. NNT is the inverse of the stratum-specific prevalence among tested adolescents

**Fig. 2:**
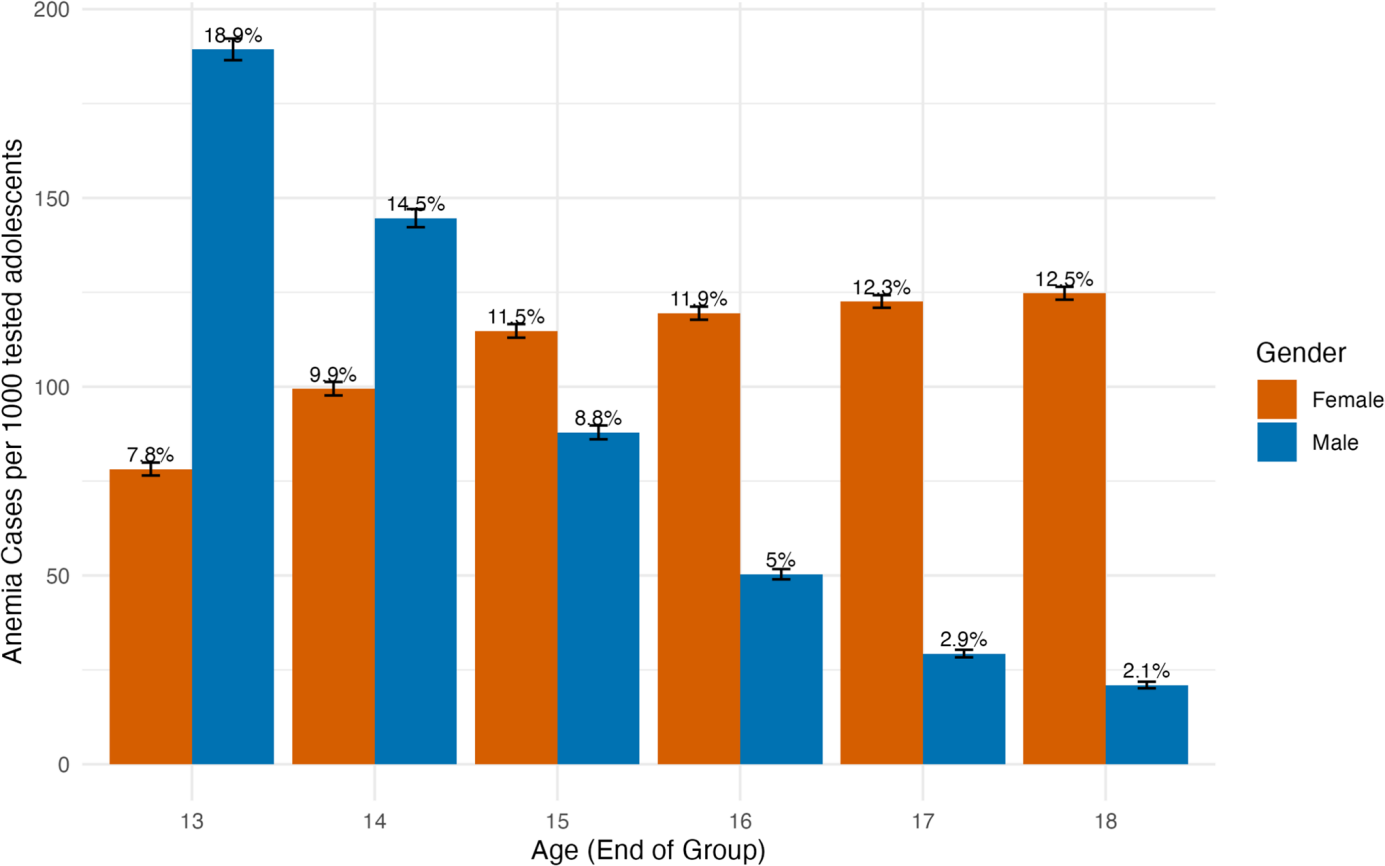
Prevalence of microcytic anemia among tested adolescents by age group and sex

Among females, prevalence increased steadily with age. This age-associated gradient was consistent across all female age groups, with the steepest relative increase occurring between ages 12 and 13 to 13 and 14 years. In contrast, among males, the prevalence of microcytic anemia was highest at younger ages and declined progressively with age (Fig. 2).

The prevalence of microcytic anemia among adolescents with recorded hemoglobin measurements is shown by age group and sex. Values are presented as percentages with 95% confidence intervals.

**Fig. 3:**
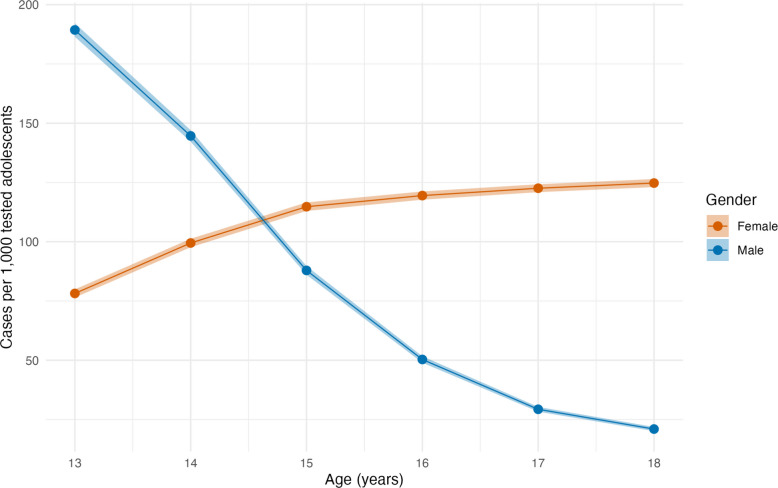
Age-specific trends in the prevalence of microcytic anemia among tested adolescents by sex

**Fig. 4:**
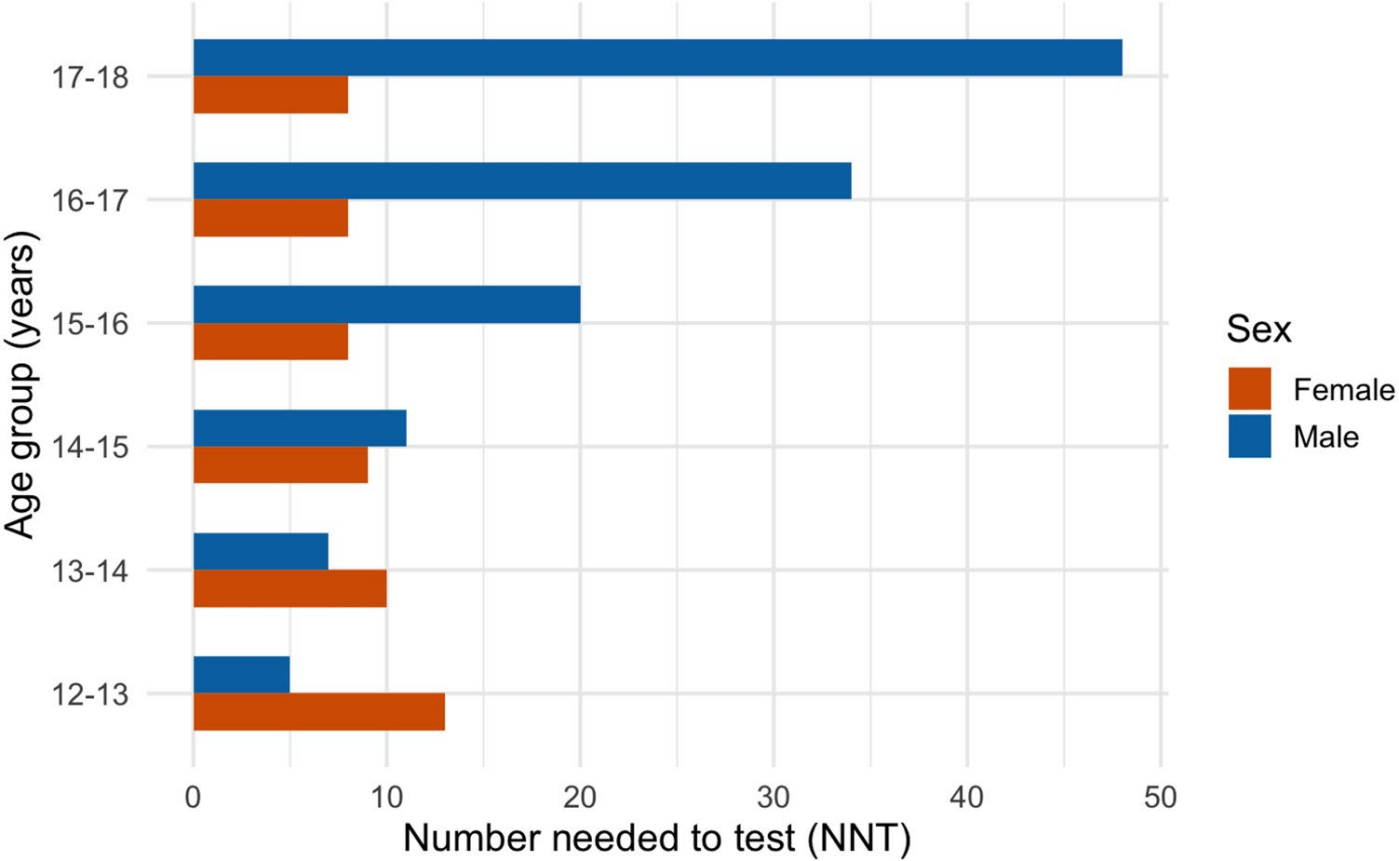
Number needed to test (NNT) to detect one case of microcytic anemia by age group and sex

The inverse age trend in males differed substantially from that observed in females, resulting in a reversal of the sex distribution of microcytic anemia prevalence during mid-adolescence, with females exceeding males from approximately age 15 onward (Fig. 3). The NNT increased markedly with age among males, indicating decreasing screening efficiency, whereas among females, NNT declined during early adolescence and remained relatively stable from ages 15–18 years (Fig. 4).

In a sensitivity analysis restricted to males aged 12–13 years, applying a lower hemoglobin threshold of < 12 g/dL reduced the estimated prevalence of microcytic anemia from 18.9% to 7.6% (95% CI, 7.4–7.8%). This was comparable to the prevalence of 7.8% (95% CI, 7.6–8.0%) observed among females of the same age.

### Proportion of microcytic anemia among the total insured population

Using a population-based denominator, the proportion of females with microcytic anemia increased with age, whereas among males, the corresponding proportions declined. Although these population-based proportions were substantially lower than the prevalence estimates among tested adolescents, the age- and sex-specific trends were similar to those observed in the primary analysis, with a progressive increase among females and a marked decline among males (Table 2).

**Table 2:** Proportion of microcytic anemia and number needed to test among the total CHS-insured adolescent population, by age group and sex

Age group (years)	Sex	Microcytic anemia cases, n	Total insured population, n	Proportion, % (95% CI)	NNT
12–13	Female	7,389	534,289	1.38 (1.35–1.41)	72.5
	Male	13,666	564,631	2.42 (2.38–2.46)	41.3
13–14	Female	10,683	525,766	2.03 (1.99–2.07)	49.3
	Male	11,831	556,353	2.13 (2.09–2.16)	46.9
14–15	Female	13,831	517,646	2.67 (2.63–2.72)	37.5
	Male	8,068	548,659	1.47 (1.44–1.50)	68.0
15–16	Female	15,982	510,309	3.13 (3.08–3.18)	31.9
	Male	4,996	541,795	0.92 (0.90–0.95)	108.7
16–17	Female	18,195	504,795	3.60 (3.55–3.66)	27.8
	Male	3,197	536,262	0.60 (0.58–0.62)	166.7
17–18	Female	17,982	499,808	3.60 (3.55–3.65)	27.8
	Male	2,175	530,184	0.41 (0.39–0.43)	243.9

*NNT* number needed to test. The denominator is the total number of adolescents insured by CHS within each age-sex stratum, regardless of whether hemoglobin testing was performed. The reported proportions represent documented microcytic anemia and should not be interpreted as true population prevalence because adolescents who were not tested could not be classified Confidence intervals were calculated using the Wilson method. NNT is the inverse of the stratum-specific population-based prevalence and represents the number of adolescents who would need to be tested to identify one case of microcytic anemia, assuming universal testing

## Discussion

This large EHR-based study evaluated age- and sex-specific patterns in the prevalence of microcytic anemia and the potential yield of screening during adolescence. Microcytic anemia prevalence increased steadily among females but declined among males, resulting in substantial differences in the expected yield of screening by age and sex.

Because ferritin measurements were not consistently available, our analysis relied on microcytic anemia rather than laboratory-confirmed IDA. Although individuals with known hereditary, hemolytic, or chronic inflammatory causes of anemia were excluded, microcytic anemia cannot be assumed to represent ID in all cases. Nevertheless, the age- and sex-specific trends observed in our data mirror the epidemiologic patterns typically reported for IDA during adolescence, allowing cautious comparison with prior studies [10, 11, 14, 16, 25].

The prevalence of IDA among adolescents varies across studies. Previous reports from developed countries describe prevalence estimates ranging from approximately 2.7% to 20% among adolescent females and from 0 to 5% among males [8, 10, 12, 13]. The National Health and Nutrition Examination Survey (NHANES) data showed that ID affected 38.6% and IDA 6.3% of females aged 12–21 years [18, 26]. Some of these estimates are comparable to or higher than those observed in the present study, whereas others report lower prevalence. Several factors likely contribute to this variability. Studies differ in the age ranges included within the adolescent population as well as in diagnostic definitions. In particular, differences in hemoglobin thresholds and the use of additional biomarkers such as ferritin may influence prevalence estimates. Population characteristics may also play an important role. Israel is characterized by considerable ethnic, socioeconomic, and cultural heterogeneity, and previous studies have demonstrated variation in anemia prevalence across population groups within the country [20, 21].

The distinct age-related trends observed between females and males likely reflect known physiologic differences in iron metabolism during adolescence. The increasing prevalence of microcytic anemia among females is generally attributed to the combined effects of rapid pubertal growth, the onset of menstruation, and increased iron requirements that may not be fully met by dietary intake [7, 27, 28]. In contrast, the declining prevalence of microcytic anemia observed among males across adolescence has been reported in other studies [16, 27] and may reflect decreasing iron requirements as growth velocity slows. The strikingly high prevalence of microcytic anemia among males aged 12–13 years in the primary analysis warrants specific comment. In the sensitivity analysis, using a hemoglobin threshold of < 12 g/dL, rather than the conventional adult male cutoff of < 13 g/dL, yielded a prevalence similar to that observed among females of the same age. This suggests that the elevated estimate in early-pubertal males largely reflects the use of an adult-derived threshold to boys who have not yet achieved adult hemoglobin concentrations, rather than a true excess of ID or other pathology. The finding is consistent with published reference data showing that hemoglobin concentrations in males rise progressively during puberty and approach adult values only in mid- to late adolescence [29].

Hemoglobin testing rates were consistently higher among females across all age groups. This pattern may reflect more frequent healthcare encounters related to menstrual concerns, a lower clinical threshold to evaluating anemia in females due to menstruation-related iron loss, and guideline recommendations emphasizing evaluation for anemia in adolescent females [14, 15, 30].

Although our study focused on microcytic anemia as a proxy for IDA, our findings further inform the evaluation of anemia screening strategies during adolescence [14, 31, 32]. We acknowledge that ID may occur in the absence of anemia and may still be associated with clinically relevant symptoms and adverse functional outcomes [33, 34]. Accordingly, future screening strategies may benefit from including assessment of ferritin levels, in addition to hemoglobin measurements, as recommended by the World Health Organization and reflected in the recent AAP clinical report, which recommends universal laboratory screening for ID in all adolescent girls who are at least 1-year post menarche, but no later than 14 years of age, using CBC and serum ferritin [19, 35]. Our findings are consistent with the rationale for screening at this age, demonstrating that the yield of detecting microcytic anemia improves by age 14 and remains relatively stable throughout later adolescence. However, because our outcome was microcytic anemia rather than ferritin-confirmed ID or IDA, these findings should be interpreted as supportive epidemiologic evidence rather than direct validation of ferritin-based screening recommendations.

This study has several strengths. The analysis was based on a large, EHR-derived dataset from a national healthcare system, reflecting real-world clinical testing patterns. The large sample size enabled age-specific estimates across adolescence, and the availability of population denominators allowed estimation of the potential yield of screening across different age groups and sexes.

This study has several limitations. Clinical indications for hemoglobin testing were unavailable, and adolescents who underwent testing may differ systematically from those who were not tested. The prevalence observed among tested individuals may therefore overestimate the true prevalence in the overall adolescent population. Differential testing rates by sex and age are particularly relevant in this context. Females may be more likely to undergo testing because of menstrual concerns, fatigue, or guideline-driven evaluation, whereas testing in younger males may reflect growth assessments or other clinical evaluations. The progressive increase in testing rates among females throughout adolescence may therefore contribute to the observed sex-specific trends. The complementary analysis using the total CHS-insured population denominator provides a conservative estimate of the proportion of adolescents with documented microcytic anemia and shows a similar age- and sex-specific pattern, but it does not rule out ascertainment bias. Ferritin measurements were not consistently available, and ID could not be directly confirmed. In addition, because the analysis was based on aggregated EHR data, we could not calculate RBC-based discriminant indices such as the Mentzer index, assess the severity distribution of microcytic anemia, including hemoglobin and MCV distributions, or the proportion of mild, moderate, and severe cases, or further distinguish IDA from thalassemia trait. Thus, the proportion of cases attributable to causes other than ID, most notably undiagnosed hemoglobinopathy traits that may not have been clinically recognized or recorded, cannot be quantified from the available data. This uncertainty is greater in ethnically heterogeneous populations, where the prevalence of the thalassemia trait may be non-negligible. Despite these limitations, the large sample size, the use of complementary denominators, the exclusion of documented hereditary and inflammatory causes of microcytic anemia, and the consistency of age- and sex-specific patterns observed across mid-adolescence support the robustness of the findings.

## Conclusions

In this large, EHR-based study, the prevalence of microcytic anemia increased among females but declined among males during adolescence. These findings suggest that targeted screening of females during mid-adolescence may be a practical strategy for identifying microcytic anemia and, by inference, its most common underlying cause, ID. In contrast, the lower and declining prevalence among males suggests limited yield from routine screening. Future studies should evaluate the cost-effectiveness and clinical impact of targeted screening strategies to better define optimal approaches for early detection and management.

## Data Availability

No datasets were generated or analysed during the current study.

## Abbreviations

CHS: Clalit Health Services
CI: Confidence interval
EHR: Electronic health records
IDA: Iron deficiency anemia
ID: Iron deficiency
MCV: Mean corpuscular volume
NNT: Numbers needed to test
